# Associations between neutrophil percentage-to-albumin ratio with all-cause and cause-specific mortality among US cancer survivors: evidence from NHANES 2005–2018

**DOI:** 10.3389/fnut.2025.1541609

**Published:** 2025-04-17

**Authors:** Mengjia Wang, Shenkangle Wang, Jiamiao Hu, Xuanxuan Wang, Yuke Pang, Xiaonan Sun

**Affiliations:** Department of Radiation Oncology, Sir Run Run Shaw Hospital, School of Medicine, Zhejiang University, Hangzhou, Zhejiang, China

**Keywords:** neutrophil percentage-to-albumin ratio, cancer survivors, mortality, prospective cohort study, NHANES, inflammation and nutrition

## Abstract

**Background:**

The neutrophil percentage-to-albumin ratio (NPAR) had been suggested as a potential prognostic biomarker in various health outcomes. However, its association with mortality in cancer survivors remains unclear.

**Methods:**

A total of 3,022 cancer survivors from the National Health and Nutrition Examination Survey (NHANES) 2005–2018 were linked to mortality outcomes from the National Death Index (NDI). Weighted Cox proportional hazards models was conducted to investigate the association between NPAR and all-cause, cancer and cardiovascular disease (CVD) mortality and the hazard ratio (HR) with 95% confidence interval (CI) were calculated. Restricted cubic spline (RCS) was used to clarify the non-linear association. Additionally, analyses for stratification and sensitivity were performed.

**Results:**

During a median follow-up of 75 months, 790 all-cause deaths occurred, including 244 from cancer and 209 from CVD. After adjustment for covariates, higher NPAR was independently associated with increased risk of all-cause mortality (HR = 1.09, 95% CI = 1.06–1.13), cancer mortality (HR = 1.05, 95% CI = 0.99–1.12), and CVD mortality (HR = 1.13, 95% CI = 1.06–1.21). The RCS revealed a U-shaped relationship for all-cause and cancer mortality, with thresholds of 12.76 and 13.60, respectively. Below the threshold, higher NPAR was associated with a reduced risk of mortality (HR = 0.90, 95% CI = 0.82–0.99; HR = 0.87, 95% CI = 0.76–0.99), whereas above the threshold, the risk of mortality increased significantly (HR = 1.14, 95% CI = 1.09–1.18; HR = 1.15, 95% CI = 1.07–1.24). Subgroup and sensitivity analyses confirmed these findings.

**Conclusion:**

The U-shaped association with all-cause and cancer mortality, along with the linear association with CVD mortality, underscores the potential of NPAR as a valuable prognostic marker in cancer survivors.

## 1 Introduction

The remarkable progress in cancer treatment had led to a steady decline in mortality rates, with 5-year relative survival rates reaching 69% between 2013 and 2019 (1, 2). This improved prognosis has, paradoxically, unveiled significant clinical challenges, as growing evidence indicates that prolonged survival renders patients more vulnerable to disease-associated chronic inflammation and nutritional depletion. Consequently, systematic evaluation of nutritional status and inflammatory markers for outcome prediction, coupled with timely therapeutic interventions, has become clinically imperative in cancer management.

The relationship between inflammation and cancer is complex. Chronic inflammation not only may impair the immune system’s ability to recognize and eliminate tumors, but also affect the prognosis of cancer patients (3, 4). Evidence indicates that inflammatory markers, including C reactive protein (CRP) (5), interleukin-6 (IL-6) (6, 7), systemic inflammatory response index (SII) (8), and neutrophil-to-lymphocyte ratio (NLR) (9), are critical factors influencing cancer prognosis. However, these established biomarkers had some limitations. CRP, as an acute-phase reactant, reflects short-term acute infection status. SII calculated as platelet count × neutrophil count / lymphocyte count, better represents inflammation and thrombogenesis, and is more commonly used as a predictive marker for coronary patients (8). In patients with nutritional risks, the Nutritional Risk Screening (NRS) 2002 score has been extensively employed in clinical settings to guide nutritional interventions (10), aiming to improve nutritional status, regulate inflammatory responses, and mitigate disease progression.

However, cancer patients frequently develop a malnutrition-inflammation complex syndrome, where nutritional depletion and chronic inflammation interact to exacerbate clinical outcomes. This pathophysiological interplay necessitates the development of integrated biomarkers that can simultaneously capture both dimensions. The neutrophil percentage-to-albumin ratio (NPAR) represents a clinically pragmatic biomarker that integrates both neutrophil percentage (reflecting systemic inflammation) and serum albumin levels (indicating nutritional status), offering a more holistic assessment of patients’ health. Compared to the neutrophil-to-albumin ratio (NAR), NPAR utilization of neutrophil percentage minimizes interference from fluid dilution effects on neutrophil concentration and incorporates information about neutrophil-lymphocyte relationships. Emerging evidence supports NPAR’s superior prognostic performance across various chronic conditions including cognitive function (11), diabetes (12), and heart failure (13), its role in predicting overall as well as specific-cause mortality in cancer patients has not been thoroughly investigated.

To bridge this gap, we employed a cohort study with a large, representative sample from the United States, analyzing the connection between NPAR and mortality from all causes and specific causes in individuals who have survived cancer. Our aim was to provide novel insights into the factors influencing long-term outcomes for cancer survivors and to identify potential opportunities for interventions that might enhance their quality of life.

## 2 Materials and methods

Our prospective cohort study utilized a representative sample from the National Health and Nutrition Examination Survey (NHANES), which was organized by the U.S. Centers for Disease Control and Prevention (CDC), to obtain data representative of the overall health and nutritional status of Americans.

The data was collected from structured interviews at participants’ homes, physical examinations at mobile centers, then laboratory analyses, employing a multistage probability sampling method. The original survey protocol underwent rigorous ethical review and received approval from the Institutional Review Board of the CDC. All participants signed an informed consent form at the start of the survey. Since the NHANES data released by the National Centers for Health Statistics (NCHS) were anonymized, ensuring privacy during analysis, no additional ethical approval or informed consent was needed of this secondary data analysis in this research.

### 2.1 Study population

This prospective cohort study included data from 12 consecutive cycles of the NHANES database from 2005 to 2018, which were further linked to mortality outcomes. Information regarding the diagnosis of malignant conditions was obtained through self-report. Participants were questioned, “Have you ever been told by a doctor or other health professional that you had cancer or a malignancy of any kind?” Those who responded in the affirmative were identified as cancer survivors, following “How old were you when cancer was first diagnosed?”

**Figure 1:**
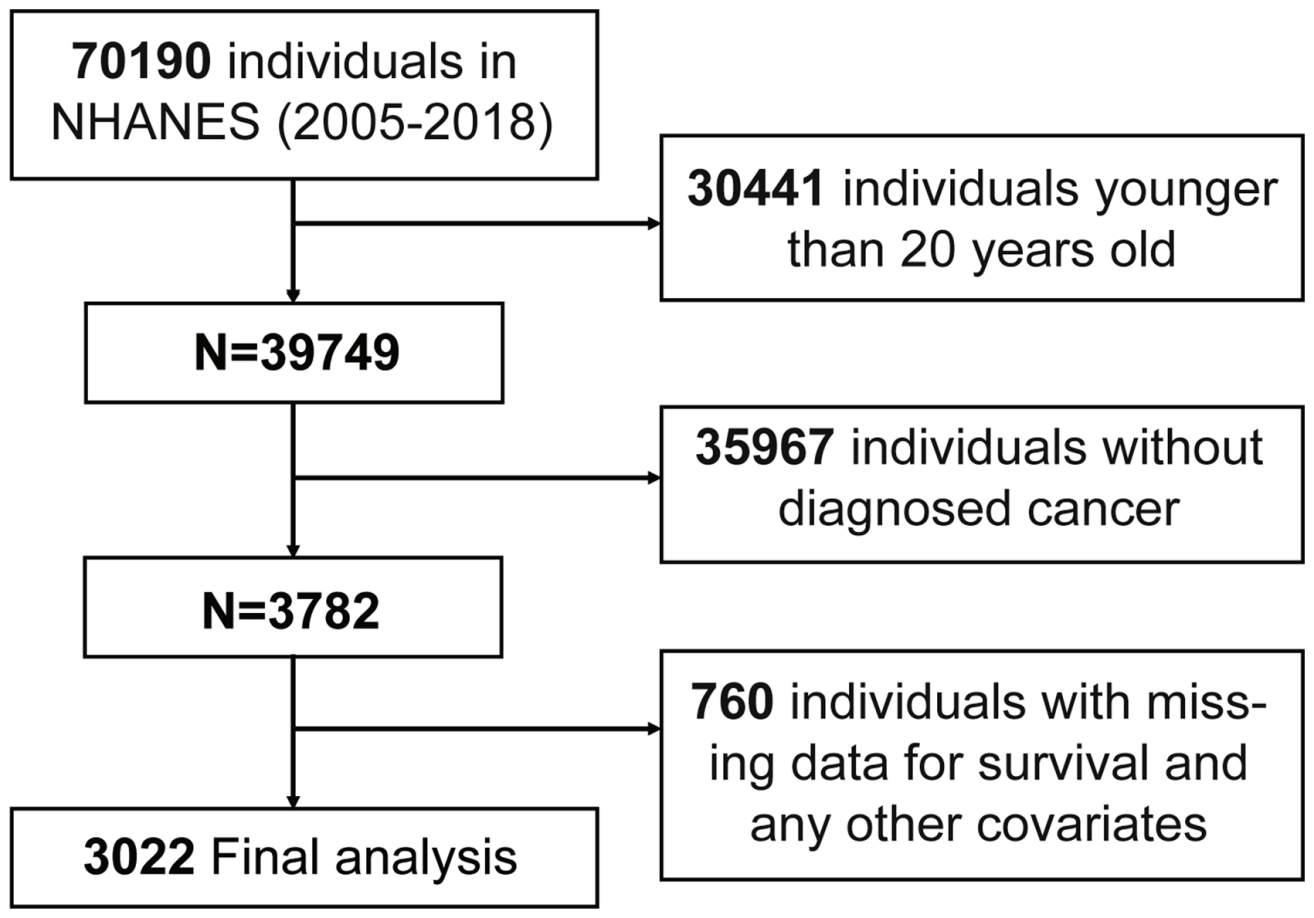
Flow chart of participants inclusion. A total of 70,190 participants from NHANES (2005–2018) were initially included. Participants were excluded due to younger age, lack of diagnosed cancer, or missing data. After exclusions, 3,022 participants were included in the final analysis. Abbreviation: NHANES, the National Health and Nutrition Examination Survey.

A total of 70,190 participates were identified from NHANES. Participants were excluded if they were under the age of 20 (*n* = 30,441) or had no cancer diagnosis (*n* = 35,967), any missing on NPAR-related test results (*n* = 490), any missing on survival and any other covariates (*n* = 270). The analysis ultimately involved 3,022 participants (Figure 1).

### 2.2 Definition of neutrophil percentage-to-albumin ratio

The Coulter^®^ HMX was used to analyze the complete blood count, while albumin was performed on the Beckman Synchron LX20 or Beckman Coulter UniCel^®^ DxC800. The NPAR was determined as follows: NPAR = neutrophil percentage (within the total white blood cell count) (%) × 100/albumin (g/dL). Detailed protocol of the lab approach is available here: https://wwwn.cdc.gov/nchs/nhanes/default.aspx.

### 2.3 Definition of mortality outcomes

To assess the survival status of the population being followed up, we linked the records from the National Death Index (NDI). All-cause mortality was defined as death from any cause. Cause-specific mortality was categorized into cardiovascular disease (CVD) or cancer-specific mortality, and these diagnoses were defined by the International Classification of Diseases, Ninth Revision (ICD-9) and Tenth Revision (ICD-10) codes according to the UCOD_LEADING code in the data dictionary. CVD-cause mortality was defined as deaths caused by diseases of heart or cerebrovascular diseases (number 001 and 005 of UCOD_LEADING). Cancer-cause mortality was defined as deaths caused by malignant neoplasms (number 002 of UCOD_LEADING). The follow-up period starts on the day of the initial interview and concludes either on the date of death or the final follow-up on December 31, 2018.

### 2.4 Covariates

Sociodemographic characteristics included age, gender (female or male), race and ethnicity (Mexican American, non-Hispanic White, non-Hispanic Black or other), marital status (married, never married, living with a partner or other), education experience (below high school, high school graduate or general equivalency or some college or above), family poverty income ratio (PIR) (under 1.3, 1.3 to 3.5 or 3.5 and above), smoke (yes or no), drink (yes or no), disease status and medication use were collected from household self-reported standardized household questionnaires. Body mass index (BMI) (under 18.5, 18.5–24.9, 25.0–29.9, or 30 and above), systolic and diastolic blood pressure were measured in the mobile examination center. Laboratory data provided measurements for total cholesterol, triglycerides, Levels of low-density lipoprotein cholesterol (LDL-C), high-density lipoprotein cholesterol (HDL-C), fasting plasma glucose, and glycohemoglobin (HbA1c). The Supplementary Data provided a comprehensive definition of concomitant diseases.

### 2.5 Statistical analyses

Since NHANES data were collected through a stratified, multistage sampling method, our analyses were all adjusted by proper weights to estimate the entire national population. Weights waves were calculated as one-seventh of subsample weights (WTMEC2YR) for 2005–2006, 2007–2008, 2009–2010, 2011–2012, 2013–2014, 2015–2016, 2017–2018 survey cycles, accounting for the fact that not all participants had blood tests. Primary sample unit (SDMVPSU) and stratum (SDMVSTRA) variables were also used in the weighting adjustment. All statistical analyses were performed using R (version 4.4.2) and R Studio (version 2024.09.1 + 394). The “svydesign” function from the “survey” package was used to create a weighted cohort, and the “svycoxph” function was employed to fit a weighted Cox proportional hazards regression model. All subsequent analyses were based on this approach. A two-sided *p* < 0.05 was considered to indicate statistical significance.

Participants were divided by the quartile of NPAR: Q1 (< 12.73), Q2 (12.73–14.42), Q3 (14.42–16.21), and Q4 (> 16.21). Continuous variables were presented as the weighted means ± standard error and categorical variables were presented as frequencies (unweighted) and percentages (weighted). Weighted Kruskal-Wallis H test, general linear model for complex samples, and weighted chi-square tests were conducted to compare both continuous variables and categorical variables. Associations between NPAR and mortality were assessed through weighted multivariate Cox regression models. In Model 1, adjustments were made for age, sex, and race. Model 2 included additional adjustments for marital status, education, PIR, BMI, smoking, and alcohol consumption. Adjustments to Model 3 accounted for all variables, like diabetes and hypertension. To examine the dose–response relationship between NPAR and mortality, a restricted cubic spline regression (RCS) with four knots and multivariable adjustment was applied. Non-linearity was assessed through the likelihood ratio test. If nonlinearity was present, the threshold was defined as the point with the lowest hazard ratios (HRs), and two-piecewise Cox regression analysis was performed. Stratified analyses were also performed by sex, race, marital status, education, PIR, BMI, smoking, drinking, diabetes, and hypertension. Several analyses of sensitivity were also executed to test the study’s robustness. First, weighted and unweighted Kaplan–Meier log-rank survival curves were performed to estimate survival between different NPAR subgroups. Second, unweighted data were entered into multivariable Cox regression to estimate hazard ratio (HR) with 95% confidence intervals (CI). Third, cancer patients who passed away within 2 years of being diagnosed were excluded.

## 3 Results

### 3.1 Baseline characteristics

A total of 3,022 cancer survivors (weighted population 19,447,868) were finally included, with an average age of 62.95 ± 14.13 years and 1,572 (56.35%) were female. Categorized by NPAR quartiles, the baseline characteristics of cancer survivors were presented in Table 1, and those related to all-cause mortality were detailed in Supplementary Table 1. Participants who had a higher NPAR were predominantly female, non-Hispanic White, and exhibited a higher BMI, and be diagnosed with diabetes and hypertension (*p* < 0.05).

**Table 1:** Basic demographic characteristics of cancer participants stratified by quartile of NPAR in NHANES 2005–2018.

Characteristic	Overall *N* = 19,447,868	Q1 *N* = 5,010,259	Q2 *N* = 5,208,861	Q3 *N* = 4,892,797	Q4 *N* = 4,335,951	*p*-value
Age	62.95 ± 14.13	60.75 ± 13.77	61.23 ± 14.05	64.04 ± 14.68	66.31 ± 13.24	<0.001
Gender						0.11
Male	1,450(43.65)	351(44.89)	353(42.36)	358(39.86)	388(48.06)	
Female	1,572(56.35)	405(55.11)	403(57.64)	395(60.14)	369(51.94)	
Race						0.025
Mexican American	199(2.49)	43(1.86)	50(2.81)	54(2.68)	52(2.60)	
Non-Hispanic White	2,096(86.87)	477(84.90)	533(87.77)	536(87.27)	550(87.60)	
Non-Hispanic Black	405(4.71)	144(6.83)	86(3.76)	86(3.67)	89(4.56)	
Other	322(5.94)	92(6.40)	87(5.65)	77(6.37)	66(5.25)	
Marital						0.028
Married	1,739(62.53)	453(65.82)	436(63.38)	430(59.54)	420(61.08)	
Never married	189(5.81)	57(7.67)	43(5.14)	52(6.21)	37(4.01)	
Living with partner	97(3.44)	29(3.74)	23(2.58)	29(4.86)	16(2.51)	
Other	997(28.22)	217(22.77)	254(28.89)	242(29.39)	284(32.40)	
Education						0.4
Below high school	614(12.04)	139(11.16)	148(10.87)	163(13.13)	164(13.21)	
High school graduate or general equivalency diploma	680(21.05)	162(18.80)	170(21.78)	174(20.53)	174(23.36)	
Some college or above	1,728(66.91)	455(70.03)	438(67.35)	416(66.35)	419(63.43)	
PIR						0.003
≤ 1.3	656(13.29)	170(12.27)	159(14.05)	152(12.28)	175(14.70)	
>1.3 to 3.5	1,381(40.92)	311(36.46)	323(36.85)	371(46.27)	376(44.93)	
>3.5	985(45.79)	275(51.26)	274(49.10)	230(41.45)	206(40.36)	
BMI						0.01
<18.5	43(1.39)	11(1.26)	12(1.57)	8(0.97)	12(1.80)	
18.5–24.9	746(26.03)	208(28.39)	195(27.85)	177(25.86)	166(21.26)	
25.0–29.9	1,052(35.11)	271(37.73)	279(37.24)	261(33.11)	241(31.72)	
≥30	1,136(37.47)	260(32.63)	263(33.34)	295(40.06)	318(45.22)	
Smoke						0.3
Yes	1,662(53.61)	372(49.84)	416(55.22)	422(53.81)	452(55.83)	
No	1,360(46.39)	384(50.16)	340(44.78)	331(46.19)	305(44.17)	
Drink						0.007
Yes	1,992(70.82)	511(74.87)	517(73.52)	503(69.24)	461(64.66)	
No	1,030(29.18)	245(25.13)	239(26.48)	250(30.76)	296(35.34)	
Diabetes						<0.001
Yes	787(21.29)	161(15.90)	175(16.82)	207(23.69)	244(30.18)	
No	2,235(78.71)	595(84.10)	581(83.18)	546(76.31)	513(69.82)	
Hypertension						<0.001
Yes	1,952(58.39)	467(52.47)	463(54.74)	494(60.01)	528(67.77)	
No	1,070(41.61)	289(47.53)	293(45.26)	259(39.99)	229(32.23)	
Hyperlipidemia						0.7
Yes	2,532(84.72)	639(85.01)	641(85.97)	632(84.13)	620(83.56)	
No	490(15.28)	117(14.99)	115(14.03)	121(15.87)	137(16.44)	

N, number of weighted populations; PIR, family poverty income ratio; BMI, body mass index.

### 3.2 Association between NPAR and mortality

During the median follow-up period of 75 months (Interquartile Range: 40–119.7), there were 790 deaths from all causes: 244 from malignant neoplasms, 209 from cardiovascular disease, and 337 from other causes. Cancer participants with a higher level of NPAR were significantly associated with an increased risk of all-cause mortality (HR = 1.16, 95% CI = 1.12–1.20), cancer-cause mortality (HR = 1.10, 95% CI = 1.03–1.17), and CVD-cause mortality (HR = 1.20, 95% CI = 1.14–1.26) (Table 2) in the unadjusted model. The results of weighted multivariate Cox regression were shown in Supplementary Table 2. After additional adjustment, the other three models continued to show robustness with respect to all-cause mortality and CVD mortality for all covariates. Cancer participants in the fourth quartile of NPAR were much higher than those in the first quartile of NPAR in model 1 (HR = 1.98, 95% CI = 1.38-2.85, *p* for trend < 0.001), model 2 (HR = 1.59, 95% CI = 1.12-2.26, *p* for trend < 0.001) (Table 2).

**Table 2:** Weighted association between NPAR and mortality and trend analysis.

	Q1	Q2	Q3	Q4	*p* for trend	NPAR
All-cause mortality						
Unadjusted	1	1.33(1.02, 1.72)	1.77(1.35, 2.33)	2.98(2.27, 3.82)	<0.001	1.16(1.12, 1.20)
Model 1	1	1.26(0.99, 1.61)	1.32(1.02, 1.71)	2.11(1.65, 2.71)	<0.001	1.11(1.08, 1.15)
Model 2	1	1.17(0.92, 1.48)	1.23(0.95, 1.59)	1.84(1.44, 2.34)	<0.001	1.09(1.06, 1.13)
Model 3	1	1.14(0.90, 1.45)	1.22(0.94, 1.58)	1.78(1.39, 2.26)	<0.001	1.09(1.06, 1.13)
Cancer mortality						
Unadjusted	1	0.86(0.57, 1.31)	1.05(0.68, 1.63)	1.98(1.38, 2.85)	<0.001	1.10(1.03, 1.17)
Model1	1	0.84(0.56, 1.27)	0.91(0.58, 1.43)	1.59(1.12, 2.26)	0.003	1.07(1.01, 1.14)
Model2	1	0.79(0.51, 1.20)	0.84(0.54, 1.31)	1.41(0.99, 2.00)	0.015	1.06(0.99, 1.12)
Model3	1	0.78(0.51, 1.19)	0.84(0.54, 1.30)	1.36(0.96, 1.95)	0.026	1.05(0.99, 1.12)
CVD mortality						
Unadjusted	1	1.88(1.09, 3.25)	3.03(1.81, 5.07)	4.77(2.95, 7.70)	<0.001	1.20(1.14, 1.26)
Model 1	1	1.70(0.99, 2.91)	1.92(1.16, 3.18)	2.99(1.83, 4.86)	<0.001	1.14(1.08, 1.21)
Model 2	1	1.57(0.89, 2.78)	1.77(1.03, 3.03)	2.68(1.59, 4.52)	<0.001	1.13(1.06, 1.21)
Model 3	1	1.48(0.84, 2.61)	1.75(1.03, 2.97)	2.56(1.52, 4.29)	<0.001	1.13(1.06, 1.21)

Data are presented as HR (95%CI), Q1: 6.79, Q2: 13.57, Q3: 15.32, Q4:26.16. Model 1: Adjusted by age, gender, race. Model 2: Adjusted by age, gender, race, marital, education, PIR, BMI, smoke, drink. Model 3: Adjusted by age, gender, race, marital, education, PIR, BMI, smoke, drink, diabetes, hypertension.

### 3.3 Non-linear relationship between NPAR and mortality

**Figure 2:**
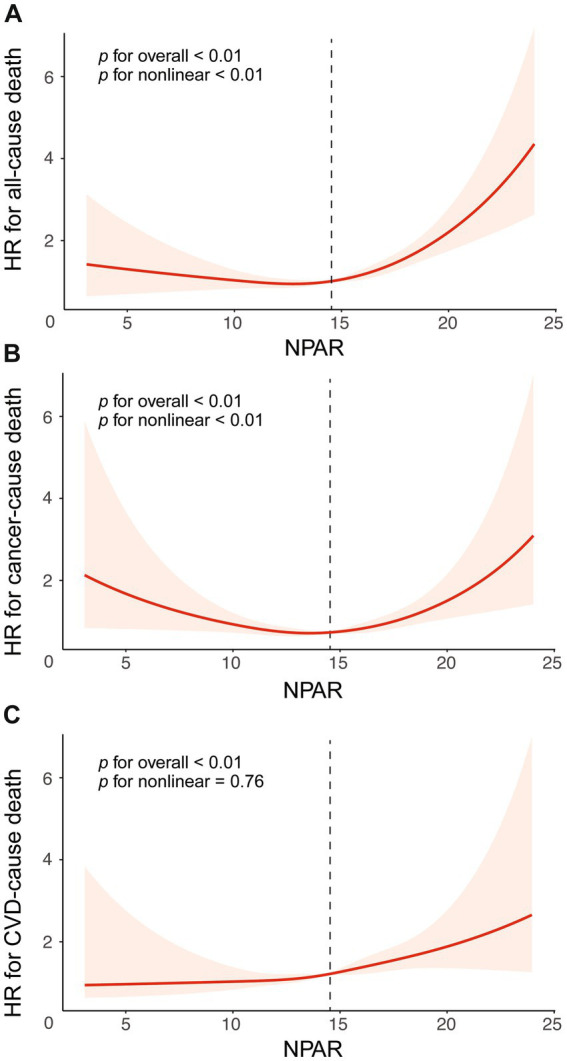
The association between NPAR and mortality risk, presented with all adjustment and 95%CI. The relationship was analyzed using restricted cubic splines for different causes of death: **(A)** All-cause mortality, **(B)** Cancer-specific mortality, and **(C)** CVD-specific mortality. Adjustments were made for age, gender, race, marital, education, PIR, BMI, smoke, drink, diabetes, hypertension. Abbreviation: 95% CI, 95% confidence intervals; HR, Hazard Ratio; NPAR, the neutrophil percentage-to-albumin ratio; CVD, cardiovascular disease.

The RCS based on adjusted weighted Cox regression model was performed to identify the non-linear relationship between NPAR and mortality from all-cause mortality (*p* < 0.01) and mortality cancer (*p* < 0.01), but not CVD mortality (*p* = 0.76). Regarding the strong U-shaped relationship between NPAR and all-cause mortality, Figure 2A illustrates a significant risk reduction down to the lowest point at NPAR value of 12.76, followed by an increase thereafter (HR = 0.90, 95% CI = 0.82–0.99, *p* = 0.02; HR = 1.14, 95% CI = 1.09–1.18, *p* < 0.001). A similar trend was shown in Figure 2B, with the risk of cancer mortality decreasing up to an NPAR value of 13.60 and then increasing with higher NPAR (HR = 0.87, 95% CI = 0.76–0.99, *p* = 0.038; HR = 1.15, 95% CI = 1.07–1.24, *p* < 0.001). The results of the two-piecewise Cox models were shown in Supplementary Table 3. However, the risk of CVD mortality increased with higher NPAR, which persisted beyond the threshold of 14.44, at which point the HR exceeded 1.0 (Figure 2C).

### 3.4 Stratified analyses and sensitivity analyses

**Figure 3:**
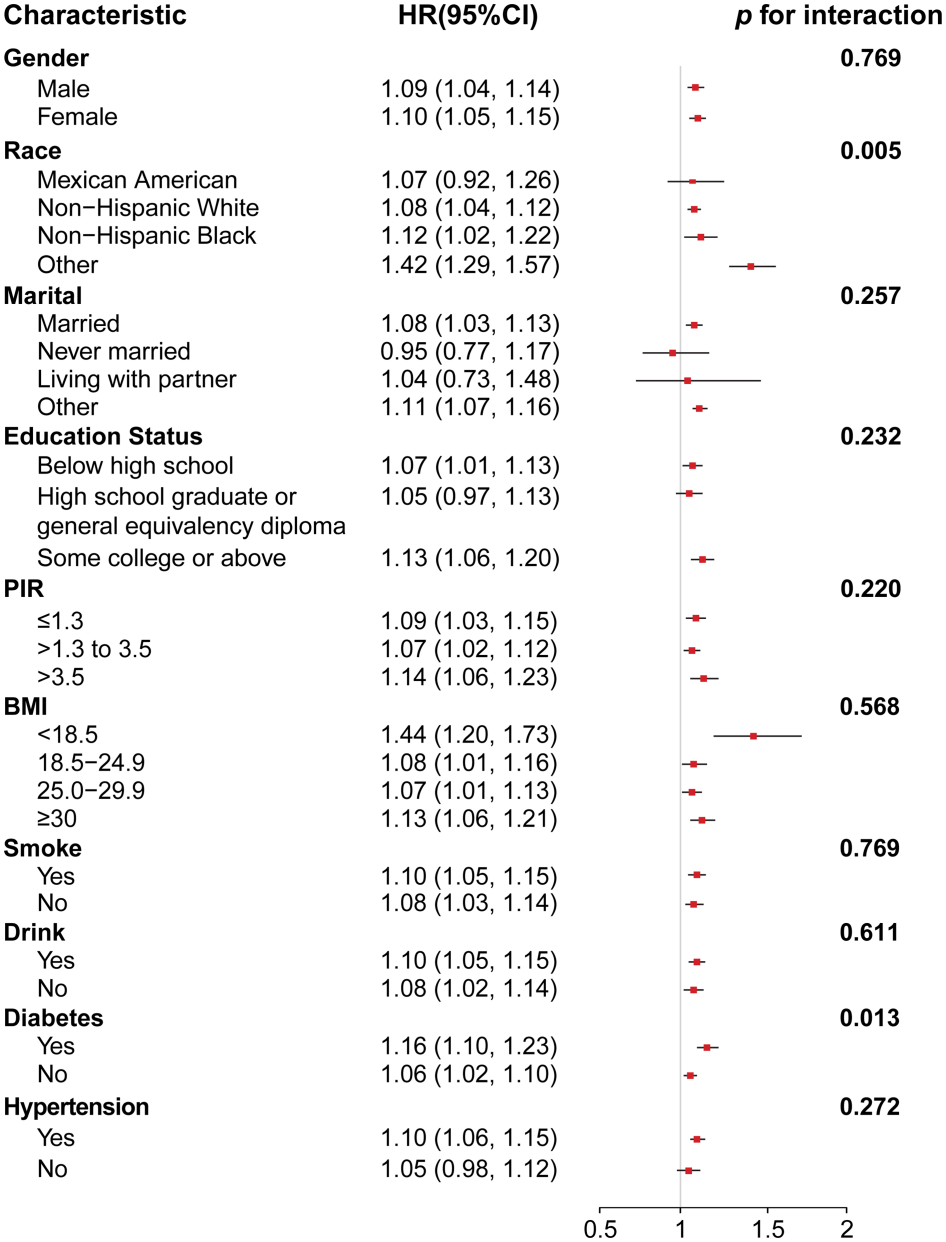
Forest plot for subgroup analysis of association between NPAR and all-cause mortality and interaction effect analysis. Hazard ratios (HRs) were calculated using weighted Cox proportional hazards regression models, adjusted for age, gender, race, marital, education, PIR, BMI, smoke, drink, diabetes, hypertension except for the stratification variables. Abbreviation: 95% CI, 95% confidence interval; HR, hazard ratio; PIR, poverty-income ratio; BMI, body mass index.

Stratified analyses were performed by sex, race, marital status, education level, PIR, BMI, smoking, alcohol consumption, diabetes, and hypertension to determine the link between NPAR and all mortality. As shown in Figure 3, consistent results were observed across subgroups by sex, married, education, PIR, BMI < 18.5 or ≥30, smoke, drink and hypertension (*p* for interaction > 0.05). However, a significant interaction was found between NPAR and race and between NPAR and diabetes (*p* for interaction < 0.05). Similar results for cancer-specific and CVD mortality are shown in Supplementary Figures 1, 2.

We also performed sensitivity analyses for the association between NPAR and mortality. First, the weighted and unweighted Kaplan–Meier survival curves (Supplementary Figures 3A–F) confirmed that cancer survivors in the fourth quartile (Q4) of NPAR had the lowest all-cause and cause-specific survival rates compared with those in the other quartiles (Q1-Q3) (*p* < 0.01). Second, the results remained consistent when unweighted data were used and when patients who died within 2 years of a cancer diagnosis were excluded (Supplementary Tables 4, 5).

## 4 Discussion

In our large, stratified, multistage study, we investigated the association between NPAR and mortality among 3,022 cancer survivors. This is the first research, to our knowledge, to reveal this relationship. This study demonstrates a U-shaped association between NPAR levels and all-cause as well as cancer-specific mortality, with both low and high NPAR levels associated with reduced survival. Conversely, there was a linear relationship between NPAR and CVD mortality, with mortality risk consistently rising as NPAR levels increased.

Higher levels of NPAR often indicate that patients are in a state of chronic inflammation and have a poor nutritional status. The impact of neutrophils on tumors is complex and diverse (14). NLRs are predictive as biomarkers of immune checkpoint response (14–16), which is associated with poor clinical prognosis in cancer patients. In some cases, neutrophils can facilitate the progression of primary tumors. In primary breast cancer, it can promote tumor progression through an IL-17-dependent pathway (17). In gastric cancer, it can lead to tumor progression by promoting T-helper cell subsets that produce tumor-promoting interleukin 17A through a series of pathways (18). However, neutrophils are not entirely associated with tumor-promoting mechanisms and there are also cases of tumor suppression (19).

Serum albumin is one of the functional proteins synthesized by the liver and has multiple functions, including the ability to protect tissues from inflammatory damage and modulate the inflammatory response (20). Decreased albumin levels not only reflect inadequate or poor absorption of nutritional intake, but can also affect the body’s immune regulation (21, 22), drug metabolism (23), and tissue repair functions (24). Therefore, this imbalance between inflammation and nutritional status may be a key factor in increased mortality (25). Several multicenter studies have validated the prognostic significance of combining body composition and systemic inflammation in cancer cachexia patients (26–28). Therefore, maintaining an adequate neutrophil percentage and a higher serum albumin level to achieve a more appropriate NPAR value will help to promote a better prognosis. Further interventional studies should be conducted to investigate whether intervening with serum albumin levels and adjusting NPAR levels in tumor patients with high levels of inflammation can improve the care and overall well-being of cancer survivors.

In stratified analysis, race is a significant influencing factor in the association between NPAR and all-cause mortality (interaction *p* = 0.005). Differences in genetic background, lifestyle and environmental factors between different races may explain this finding. For example, some races may have a stronger genetic predisposition to inflammatory responses (29, 30), and changes in NPAR may have a greater impact on mortality in these populations. In people with diabetes, the association between NPAR and all-cause mortality is stronger (*p* for interaction = 0.013). Diabetes tends to cause chronic inflammation, which can lead to increased levels of inflammatory factors in the body (31), which in turn increases neutrophil activation and affects NPAR (32). A study analyzing the mortality rate of diabetics from 1988 to 2018 also found similar results (33), but these studies usually combine diabetics with controlled blood sugar and those without effective blood sugar control for analysis, which may also introduce some bias. Nevertheless, such results are convincing. In cancer patients with diabetes, more attention should be paid to their NPAR, and timely interventions should be taken to regulate inflammation and nutritional status, which may help improve prognosis.

There are several limitations in our study. For example, the NHANES database does not provide information regarding neoadjuvant or adjuvant therapies, which have impact on prognosis of patients. Notably, another limitation from our study is that the cancer diagnoses from NHANES are self-reported and may cause some biases.

The interaction between inflammation and nutrition reflected by NPAR is complex, especially in the context of cancer. Studies have revealed that neutrophil infiltration can promote carcinogenesis by inducing IL-6 expression (34). Another research shows that TNF from neutrophils can result in immune suppression and therapy resistance in pancreatic cancer (35). So, neutrophils can influence tumor progression via inflammatory cytokines. However, these cytokines can also impact patients’ albumin levels. For instance, in liver tumors, IL-6 and TNF from Kupffer cells can regulate hepatic albumin synthesis (36). Thus, NPAR, as a composite indicator, might mirror the inflammatory factors and related internal mechanisms in the tumor microenvironment. It could better represent a patient’s inflammation and nutrition status and may serve as a tumor prognosis indicator.

In clinical practice, neutrophil percentage and albumin are easily accessible, inexpensive, and convenient to measure, and the calculation of their ratio is also very intuitive and convenient, which can provide a quick risk assessment reference for long-term follow-up of tumor patients. Nonetheless, there are still some limitations in our study. Currently, studies on NPAR are mainly observational studies, and there is a lack of interventional research evidence to support improving patient prognosis by adjusting NPAR. Therefore, long-term longitudinal studies should be conducted to dynamically monitor changes in NPAR in cancer survivors and determine its key time points in disease progression, treatment response, and prognosis assessment. At the same time, by means of diet adjustment, exercise intervention, anti-inflammatory treatment, etc., to adjust NPAR levels and explore its impact on the mortality rate and quality of life of cancer survivors, to provide evidence-based intervention strategies for clinical practice.

## 5 Conclusion

Our findings suggested that NPAR had U-shaped non-linear association with all-cause and cancer mortality, and linearly association with CVD mortality. NPAR was supported to be an independent risk factor for all-cause and specific-cause mortality in cancer survivors. Based on distinct NPAR thresholds and the multiple concurrent risks faced by patients, we recommend maintaining NPAR within the range of 12.76 to 13.60 to minimize all-cause and cause-specific mortality risks. Clinical interventions targeting nutrition and systemic inflammation should be implemented when NPAR deviates significantly from this optimal interval.

## Data Availability

Publicly available datasets were analyzed in this study. This data can be found at: https://www.cdc.gov/nchs/nhanes/.
