# Supplementary material for: Associations between neutrophil percentage-to-albumin ratio with all-cause and cause-specific mortality among US cancer survivors: evidence from NHANES 2005–2018
Source: Front Nutr. 2025 Apr 17;12:1541609. doi: 10.3389/fnut.2025.1541609 (PMC12044878; doi:10.3389/fnut.2025.1541609)
Supplement: Supplementary file 1 [file Table_1.docx]

Supplementary Material

# Supplementary Data

Diagnosed with Diabetes if they:

(1) self-reported “yes” in “have you ever been told by a doctor or health professional that diabetes or sugar diabetes”, “{Is SP/Are you} now taking insulin”, “{Is SP/Are you} now taking diabetic pills to lower {{his/her}/your} blood sugar? These are sometimes called oral agents or oral hypoglycemic agents” of Questionnaires.

(2) Glycohemoglobin HbA1c > 6.5%.

(3) Plasma Fasting Glucose ≥ 126 mg/dL.

Diagnosed with Hypertension if they:

(1) self-reported “yes” in “{Have you/Has SP} ever been told by a doctor or other health professional that {you/s/he} had hypertension, also called high blood pressure?”, “Because of {your/SP's} (high blood pressure/hypertension), {have you/has s/he} ever been told to . . . take prescribed medicine?” of Questionnaires.

(2) average Diastolic Blood Pressure > 90 mmHg or average Systolic Blood Pressure >140 mmHg.

Diagnosed with Hyperlipidemia if they:

(1) Triglyceride > 150mg/dl.

(2) Total Cholesterol > 200mg/dl

(3) LDL ≥ 130mg/dl.

(4) Low HDL: HDL < 40mg/dl (male),50mg/dl (female).

# Supplementary Tables

## Supplementary Table 1. Baseline characteristics

| **Characteristic** | **Overall N = 19,447,868** | **Alive** | **Dead** | ***p* -value** |
| --- | --- | --- | --- | --- |
| Age | 62.95±14.13 | 60.60±13.97 | 73.23±9.47 | <0.001 |
| Gender |  |  |  | <0.001 |
| Male | 1,450(43.65) | 981(41.48) | 469(53.16) |  |
| Female | 1,572(56.35) | 1,251(58.52) | 321(46.84) |  |
| Race |  |  |  | <0.001 |
| Mexican American | 199(2.49) | 174(2.78) | 25(1.21) |  |
| Non-Hispanic White | 2,096(86.87) | 1,474(86.20) | 622(89.78) |  |
| Non-Hispanic Black | 405(4.71) | 297(4.40) | 108(6.05) |  |
| Other | 322(5.94) | 287(6.62) | 35(2.95) |  |
| Marital |  |  |  | <0.001 |
| Married | 1,739(62.53) | 1,332(64.76) | 407(52.77) |  |
| Never married | 189(5.81) | 153(6.12) | 36(4.46) |  |
| Living with partner | 97(3.44) | 84(3.94) | 13(1.22) |  |
| Other | 997(28.22) | 663(25.18) | 334(41.55) |  |
| Education |  |  |  | <0.001 |
| Below high school | 614(12.04) | 373(9.35) | 241(23.80) |  |
| High school graduate or general equivalency diploma | 680(21.05) | 489(19.87) | 191(26.20) |  |
| Some college or above | 1,728(66.91) | 1,370(70.78) | 358(50) |  |
| PIR |  |  |  | <0.001 |
| ≤ 1.3 | 656(13.29) | 456(11.71) | 200(20.22) |  |
| >1.3 to 3.5 | 1,381(40.92) | 968(38.33) | 413(52.26) |  |
| >3.5 | 985(45.79) | 808(49.96) | 177(27.52) |  |
| BMI |  |  |  | <0.001 |
| <18.5 | 43(1.39) | 22(0.93) | 21(3.43) |  |
| 18.5–24.9 | 746(26.03) | 521(25.37) | 225(29.01) |  |
| 25.0-29.9 | 1,052(35.11) | 777(34.96) | 275(35.79) |  |
| ≥30 | 1,136(37.47) | 892(38.74) | 244(31.77) |  |
| Smoke |  |  |  | <0.001 |
| Yes | 1,662(53.61) | 1,165(51.46) | 497(63.03) |  |
| No | 1,360(46.39) | 1,067(48.54) | 293(36.97) |  |
| Drink |  |  |  | 0.016 |
| Yes | 1,992(70.82) | 1,475(71.98) | 517(65.72) |  |
| No | 1,030(29.18) | 757(28.02) | 273(34.28) |  |
| Diabetes |  |  |  | <0.001 |
| Yes | 787(21.29) | 529(19.15) | 258(30.68) |  |
| No | 2,235(78.71) | 1,703(80.85) | 532(69.32) |  |
| Hypertension |  |  |  | <0.001 |
| Yes | 1,952(58.39) | 1,347(54.29) | 605(76.31) |  |
| No | 1,070(41.61) | 885(45.71) | 185(23.69) |  |
| Hyperlipidemia |  |  |  | 0.5 |
| Yes | 2,532(84.72) | 1,884(84.99) | 648(83.56) |  |
| No | 490(15.28) | 348(15.01) | 142(16.44) |  |

**Abbreviation**: PIR, family poverty income ratio; BMI, body mass index.

## Supplementary Table 2. Multivariate analysis of COX regression model

| **Characteristic** | **HR** | **95% CI** | ***p* -value** |
| --- | --- | --- | --- |
| NPAR | 1.09 | 1.06, 1.13 | <0.001 |
| Age | 1.07 | 1.04, 1.09 | <0.001 |
| Gender |  |  |  |
| Male | 1 | 1 |  |
| Female | 0.66 | 0.53,0.83 | <0.001 |
| Race |  |  |  |
| Mexican American | 1 | 1 |  |
| Non-Hispanic White | 1,47 | 1.04,2.06 | 0.028 |
| Non-Hispanic Black | 1.53 | 1.04,2.21 | 0.029 |
| Other | 1.05 | 0.64,1.73 | 0.8 |
| Marital |  |  |  |
| Married | 1 | 1 |  |
| Never married | 1.68 | 1.04,2.70 | 0.032 |
| Living with partner | 1.01 | 0.55,1.85 | >0.9 |
| Other | 1.55 | 1.32,1.83 | <0.001 |
| Education |  |  |  |
| Below high school | 1 | 1 |  |
| High school graduate or general equivalency diploma | 0.92 | 0.73,1.16 | 0.5 |
| Some college or above | 0.74 | 0.58,0.94 | 0.015 |
| PIR |  |  |  |
| ≤ 1.3 | 1 | 1 |  |
| >1.3 to 3.5 | 0.64 | 0.50,0.82 | <0.001 |
| >3.5 | 0.51 | 0.37,0.68 | <0.001 |
| BMI |  |  |  |
| <18.5 | 1 | 1 |  |
| 18.5–24.9 | 0.44 | 0.26,0.76 | 0.003 |
| 25.0-29.9 | 0.32 | 0.19,0.55 | <0.001 |
| ≥30 | 0.33 | 0.19,0.57 | <0.001 |
| Smoke |  |  |  |
| Yes | 1 | 1 |  |
| No | 0.68 | 0.56,0.83 | <0.001 |
| Drink |  |  |  |
| Yes | 1 | 1 |  |
| No | 1.23 | 1.00,1.51 | 0.052 |
| Diabetes |  |  |  |
| Yes | 1 | 1 |  |
| No | 0.71 | 0.57,0.88 | 0.002 |
| Hypertension |  |  |  |
| Yes | 1 | 1 |  |
| No | 0.79 | 0.65, 0.96 | 0.015 |

**Abbreviation**: NPAR, neutrophil percentage-to-albumin ratio; PIR, family poverty income ratio; BMI, body mass index.

## Supplementary Table 3. Nonlinearity addressed through two-piecewise Cox model.

|  | **All-cause mortality^a^** | **P-value** | **Cancer mortality^a^** | ***p* -value** |
| --- | --- | --- | --- | --- |
| **Threshold value^b^** | 12.76 |  | 13.6 |  |
| < | 0.90(0.82,0.99) | 0.02 | 0.87(0.76,0.99) | 0.038 |
| ≥ | 1.14(1.09,1.18) | <0.001 | 1.15(1.07,1.24) | <0.001 |

^a^ Hazard ratios were calculated by multivariable Cox proportional hazards regression model Adjusted for age, gender, race, marital, education, PIR, BMI, smoke, drink, diabetes, hypertension.

^b^ Threshold values were 12.76 and 13.60 for all-cause and cancer mortality respectively.

## Supplementary Table 4. Hazard ratios (95% CI) of all-cause and specific cause mortality according to quartiles of NPAR among cancer patients

|  | **Q1** | **Q2** | **Q3** | **Q4** | ***p* for Trend** | **NPAR** |
| --- | --- | --- | --- | --- | --- | --- |
| All-cause Mortality |  |  |  |  |  |  |
| Unadjusted | 1 | 1.29(0.96,1.73) | 1.62(1.20,2.19) | 2.60(1.91,3.53) | <0.001 | 1.13(1.08,1.18) |
| Model1 | 1 | 1.21(0.92,1.61) | 1.17(0.87,1.56) | 1.85(1.37,2.49) | <0.001 | 1.09(1.04,1.13) |
| Model2 | 1 | 1.11(0.84,1.46) | 1.08(0.81,1.46) | 1.60(1.10,2.14) | <0.001 | 1.07(1.02,1.11) |
| Model3 | 1 | 1.09(0.82,1.44) | 1.08(0.81,1.46) | 1.56(1.16,2.09) | 0.002 | 1.06(1.02,1.11) |
| Cancer Mortality |  |  |  |  |  |  |
| Unadjusted | 1 | 0.87(0.51,1.46) | 0.88(0.52,1.50) | 1.88(1.21,2.93) | 0.009 | 1.06(0.98,1.15) |
| Model1 | 1 | 0.83(0.50,1.38) | 0.72(0.42,1.22) | 1.47(0.94,2.29) | 0.1 | 1.02(0.94,1.11) |
| Model2 | 1 | 0.74(0.44,1.24) | 0.66(0.39,1.10) | 1.26(0.80,1.98) | 0.3 | 1.01(0.93,1.09) |
| Model3 | 1 | 0.74(0.44,1.24) | 0.66(0.39,1.09) | 1.23(0.77,1.95) | 0.3 | 1.00(0.92,1.09) |
| CVD Mortality |  |  |  |  |  |  |
| Unadjusted | 1 | 1.65(0.91,2.97) | 2.48(1.46,4.20) | 3.63(2.19,6.02) | <0.001 | 1.17(1.11,1.24) |
| Model1 | 1 | 1.48(0.82,2.68) | 1.54(0.91,2.62) | 2.30(1.36,3.89) | <0.001 | 1.11(1.04,1.19) |
| Model2 | 1 | 1.37(0.74,2.56) | 1.48(0.85,2.60) | 2.15(1.23,3.75) | <0.001 | 1.10(1.03,1.18) |
| Model3 | 1 | 1.30(0.70,2.41) | 1.47(0.85,2.56) | 2.06(1.18,3.59) | 0.003 | 1.10(1.02,1.18) |

Data are presented as HR (95%CI), Q1: 6.78, Q2: 13.54, Q3: 15.25, Q4:22.10.

Model 1: Adjusted by age, gender, race.

Model 2: Adjusted by age, gender, race, marital, education, PIR, BMI, smoke, drink.

Model 3: Adjusted by age, gender, race, marital, education, PIR, BMI, smoke, drink, diabetes, hypertension.

## Supplementary Table 5. Hazard ratios (95% CI) of all-cause and specific cause mortality according to quartiles of NPAR among unweighted cancer

|  | **Q1** | **Q2** | **Q3** | **Q4** | ***p* for Trend** | **NPAR** |
| --- | --- | --- | --- | --- | --- | --- |
| All-cause Mortality |  |  |  |  |  |  |
| Unadjusted | 1 | 1.24(0.99,1.55) | 1.65(1.22,2.04) | 2.39(1.95,2.93) | <0.001 | 1.13(1.10,1.15) |
| Model1 | 1 | 1.11(0.88,1.39) | 1.26(1.02,1.57) | 1.88(1.53,2.31) | <0.001 | 1.10(1.08,1.13) |
| Model2 | 1 | 1.05(0.84,1.32) | 1.20(0.97,1.50) | 1.69(1.36,2.08) | <0.001 | 1.09(1.06,1.12) |
| Model3 | 1 | 1.04(0.83,1.30) | 1.20(0.96,1.50) | 1.65(1.34,2.04) | <0.001 | 1.09(1.06,1.12) |
| Cancer Mortality |  |  |  |  |  |  |
| Unadjusted | 1 | 0.88(0.60,1.28) | 1.01(0.70,1.46) | 1.57(1.12,2.20) | 0.002 | 1.07(1.02,1.11) |
| Model1 | 1 | 0.83(0.57,1.20) | 0.87(0.60,1.27) | 1.33(0.95,1.88) | 0.031 | 1.05(1.00,1.10) |
| Model2 | 1 | 0.79(0.54,1.15) | 0.82(0.56,1.18) | 1.20(0.85,1.70) | 0.12 | 1.03(0.98,1.08) |
| Model3 | 1 | 0.78(0.53,1.14) | 0.81(0.56,1.18) | 1.17(0.81,1.66) | 0.2 | 1.03(0.98,1.08) |
| CVD Mortality |  |  |  |  |  |  |
| Unadjusted | 1 | 1.61(0.99,2.63) | 2.55(1.61,4.03) | 3.72(2.39,5.79) | <0.001 | 1.17(1.12,1.22) |
| Model1 | 1 | 1.38(0.84,2.25) | 1.71(1.08,2.70) | 2.69(1.72,4.19) | <0.001 | 1.15(1.09,1.21) |
| Model2 | 1 | 1.32(0.80,2.19) | 1.63(1.01,2.62) | 2.42(1.53,3.84) | <0.001 | 1.13(1.07,1.20) |
| Model3 | 1 | 1.27(0.76,2.10) | 1.60(1.00,2.58) | 2.35(1.48,3.74) | <0.001 | 1.13(1.07,1.20) |

Data are presented as HR (95%CI), Q1: 6.78, Q2: 13.54, Q3: 15.25, Q4:22.10.

Model 1: Adjusted by age, gender, race.

Model 2: Adjusted by age, gender, race, marital, education, PIR, BMI, smoke, drink.

Model 3: Adjusted by age, gender, race, marital, education, PIR, BMI, smoke, drink, diabetes, hypertension.

# Supplementary Figures

## Supplementary Figure 1. Forest plot for subgroup analysis of association between NPAR and cancer-cause mortality

Forest plot for subgroup analysis of association between NPAR and cancer-cause mortality and interaction effect analysis. Hazard ratios (HRs) were calculated using weighted Cox proportional hazards regression models, adjusted for age, gender, race, marital, education, PIR, BMI, smoke, drink, diabetes, hypertension except for the stratification variables.

**Abbreviation:** 95% CI, 95% confidence interval; HR, hazard ratio; PIR, poverty-income ratio; BMI, body mass index.

## Supplementary Figure 2. Forest plot for subgroup analysis of association between NPAR and CVD mortality.

Forest plot for subgroup analysis of association between NPAR and CVD-cause mortality and interaction effect analysis. Hazard ratios (HRs) were calculated using weighted Cox proportional hazards regression models, adjusted for age, gender, race, marital, education, PIR, BMI, smoke, drink, diabetes, hypertension except for the stratification variables.

**Abbreviation:** 95% CI, 95% confidence interval; HR, hazard ratio; PIR, poverty-income ratio; BMI, body mass index.

## Supplementary Figure 3. Kaplan-Meier curves showing the association between NPAR and survival.

Kaplan-Meier survival curves and log-rank analyses were used to compare survival probabilities.

**Abbreviation:** CVD, cardiovascular disease.
